# Natural killer cells modified with a Gpc3 aptamer enhance adoptive immunotherapy for hepatocellular carcinoma

**DOI:** 10.1007/s12672-023-00780-6

**Published:** 2023-09-04

**Authors:** Youshi Zheng, Zisen Lai, Bing Wang, Zuwu Wei, Yongyi Zeng, Qiuyu Zhuang, Xiaolong Liu, Kecan Lin

**Affiliations:** 1 Liver Disease Center, The First Affiliated Hospital of Fujian Medical University, Fuzhou, 350005 China; 2 The United Innovation of Mengchao Hepatobiliary Technology Key Laboratory of Fujian Province, Mengchao Hepatobiliary Hospital of Fujian Medical University, Fuzhou, People’s Republic of China; 3 Mengchao Med-X Center, Fuzhou University, Fuzhou, People’s Republic of China; 4 Fujian Provincial Clinical Research Center for Hepatobiliary and Pancreatic Tumors, Fuzhou, People’s Republic of China

**Keywords:** Natural killer cells, Gpc3 DNA aptamer, Click chemistry, Glycometabolism, Hepatocellular carcinoma, Adoptive immunotherapy

## Abstract

**Introduction:**

Natural killer cells can attack cancer cells without prior sensitization, but their clinical benefit is limited owing to their poor selectivity that is caused by the lack of specific receptors to target tumor cells. In this study, we aimed to endow NK cells with the ability to specifically target glypican-3^+^ tumor cells without producing cell damage or genetic alterations, and further evaluated their therapeutic efficiency.

**Methods:**

NK cells were modified with a Gpc3 DNA aptamer on the cell surface via metabolic glycoengineering to endow NK cells with specific targeting ability. Then, the G-NK cells were evaluated for their specific targeting properties, cytotoxicity and secretion of cytokines in vitro. Finally, we investigated the therapeutic efficiency of G-NK cells against glypican-3^+^ tumor cells in vivo.

**Results:**

Compared with NK cells modified with a random aptamer mutation and unmodified NK cells, G-NK cells induced significant apoptosis/necrosis of GPC3^+^ tumor cells and secreted cytokines to preserve the intense cytotoxic activities. Moreover, G-NK cells significantly suppressed tumor growth in HepG2 tumor-bearing mice due to the enhanced enrichment of G-NK cells at the tumor site.

**Conclusions:**

The proposed strategy endows NK cells with a tumor-specific targeting ability to enhance adoptive therapeutic efficiency in GPC3^+^ hepatocellular carcinoma.

**Supplementary Information:**

The online version contains supplementary material available at 10.1007/s12672-023-00780-6.

## Introduction

Adoptive cell therapy (ACT) based on natural killer cells (NK cells) is a promising strategy in the clinic, as NK cells can induce antigen-independent immune responses [1, 2]. In addition, due to their important role in the innate immune system, NK cells also play a vital role in antitumor immune responses [3]. Activated NK cells recognize tumor cells by “missing-self” recognition or “stress-induced recognition” instead of expressing antigen-specific T cell receptors [4, 5], after which NK cells induce the death of malignant cells through direct (releasing cytotoxic granules) or indirect (activating intracellular cell death pathways) patterns [6]. These unique, rapid effects and the absence of preimmunization confer NK cells with great potential in adaptive immunotherapy [7]. However, the lack of cell specific receptors for tumor cells hinders the clinical efficacy of NK cells [8]. Recently, genetic engineering of NK cells with chimeric antigen receptor (CAR NK cells) to enhance specific targeting properties has received extensive clinical attention [9–11] since it showed promising therapeutic efficiency against different cancer types [12]. However, the generation of CAR NK cells still faces great challenges, such as the risk of transgene insertional mutagenesis [13], complex operation procedures and high costs with long-term use [14–16]. Therefore, developing a safe, simple and general technology to engineer NK cells with tumor-specific targeting properties is urgently needed.

Aptamers are single-stranded oligonucleotides with unique three dimensional structures that can recognize specific targets, such as proteins, nucleic acids or cells, with high affinity [17–19]. Moreover, aptamers have several advantages such as synthetic accessibility, low toxicity, flexible chemical modifications and a lack of immunogenicity [20, 21]. Recently, aptamers were screened by systematic evolution of ligands by exponential enrichment (SELEX) to specifically target tumor cells to enhance cancer immunotherapy [22, 23]. For instance, Dong et al. screened an APS613-1 aptamer to specifically bind to the GPC3 protein by capillary electrophoresis-SELEX technology [24, 25]; Yang et al. verified that aptamers could be anchored on the surface of immune cells to endow immune effector cells with tumor specific targeting properties [20]; Zhang et al. equipped NK cells with TLS11a and PD-L1 aptamers to enhance adoptive immunotherapy in solid tumor [26]. Given these unique features, the use of aptamers provides a new avenue for immune cells to target tumor cells and enhance the therapeutic efficacy of ACT.

**Scheme 1:**
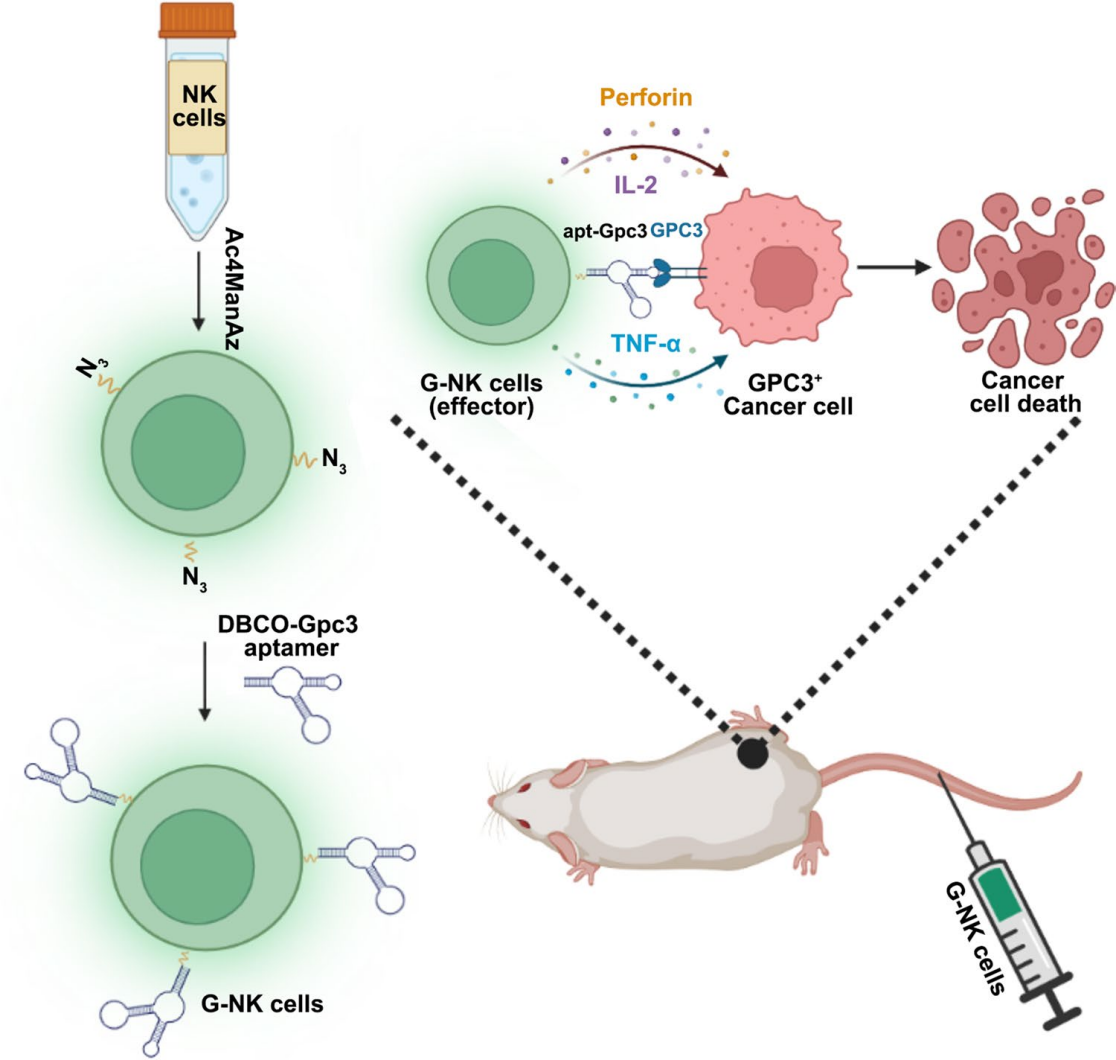
Schematic illustration of the G-NK cells to enhance adoptive immunotherapeutic efficiency of NK cells in GPC3^+^ solid tumors. *i.v* injection of G-NK cells can enhance the intercellular interaction between NK cells and GPC3^+^ tumors cells through the specific targeting property of G-NK cells

Hepatocellular carcinoma (HCC) tumors are among the most common types of solid tumors, and approximately 56.3% of HCC overexpress the GPC3 protein in serum [27]. Improving the specific targeting properties of NK cells toward the GPC3 protein can effectively improve the therapeutic effect of ACT in GPC3 positive HCC. In this study, we developed artificial engineered G-NK cells without genetic modification for cancer immunotherapy (Scheme 1). To obtain tumor cell specific targeting properties, Gpc3 aptamers were used to modify the surface of NK cells (G-NK cells) by metabolic glycan engineering without genetic alteration. Under the guidance of the surface-modified aptamer, the G-NK cells could bind to GPC3^+^ tumor cells and subsequently trigger cell apoptosis and necrosis in vitro, leading to enhanced immunotherapeutic efficiency in vivo after enrichment at the tumor site*.* These exciting results open a powerful avenue and hold considerable potential for adoptive cell therapy against HCC.

## Methods

### Chemical and reagents

An Annexin V-APC/Propidine iodide staining kit was purchased from Dojindo Co., Ltd (Cat. No: AD11, Shanghai, China). Anti-human CD3, anti-human CD16 and anti-human CD56 antibodies were obtained from eBioscience (Cat. No: 11-0039-42, 17-0168-42, 17–0567-42, San Diego, CA, USA). DI-water was obtained from a Milli-Q Gradient System (Millipore, Bedford MA, USA). Lymphocyte serum-free medium (KBM581 Medium) was purchased from Corning (Cat. No: 88-581-CM, New York, USA). Indocyanine green was obtained from DanDong YiChuang Pharmaceutical Co., Ltd. (SFDA approval Number: H20073073, Liaoning, China). Recombinant human IL-2 was obtained from Kingsley Pharmaceutical Co., Ltd. (SFDA approval Number: S10970056, Jiangshu, China). The Gpc3 aptamer was synthesized and purified by HPLC by Sangon Biotech (Shanghai, China). ELISA kits for IFN-γ, TNF-α, perforin and IL-2 were purchased from Neobioscience Technology (Cat. Nos: IFN-γ, EHC102g.96; TNF-α, EHC103a.96; Perforin, EHC154.96; IL-2, EHC003(H) 0.96; Shenzhen, China). The LDH cytotoxicity assay kit was purchased from Beyotime Biotechnology (Cat. No: C0016, Shanghai, China). The Hematoxylin–Eosin (HE) stain kit was purchased from Solarbio life sciences (Cat. No: G1120, Beijing, China).

### Cell lines and animals

HepG2 and HeLa cells were obtained from ATCC and cultured in DMEM supplemented with 10% FBS and 1% penicillin–streptomycin at 37 ℃ in a humidified incubator containing 5% CO_2_. Mature human NK cells were obtained in KBM581 containing 1000 IU/mL rhIL-2 according to our previously reported procedure [28] and verified by staining with CD3^−^/CD56^+^ and CD3^−^/CD16^+^ antibodies respectively. BALB/c nude mice (20–22 g) obtained from Wushi, Inc. (Shanghai, China) were divided to four groups: PBS group, NK cell-treated group, R-NK cell-treated group and G-NK cell-treated group (n = 3 for the in vivo distribution experiment and n = 4 for the therapeutic experiment). All animal procedures were approved by the Animal Ethics Committee of Mengchao Hepatobiliary Hospital of Fujian Medical University.

### *Preparation and characterizationof G-NK cells*

To obtain G-NK cells, activated NK cells were cultured in KBM 581 medium containing 50 μM Ac_4_ManNAz for 48 h and then washed with DPBS 3 times. Afterward, the NK cells were further modified with the DBCO-Gpc3 aptamer in DPBS for 30 min at 37 °C to obtain G-NK cells.

To evaluate the influence of incubation with Ac4ManNAz and DBCO-Gpc3 on NK cells, activated NK cells were cultured with 50 μM Ac_4_ManNAz for 24 h or 48 h. Then, the viability of NK cells and Ac_4_ManNAz treated NK cells was analyzed by CCK8 and Annexin-V/PI assays. In addition, the viability of the obtained G-NK cells and NK cells after in vitro culture for another 24 h or 48 h was also analyzed by CCK8 and Annexin-V/PI assays. ${\text{The}} {\text{relative}} {\text{fluorescent of}} {\text{G - NK}}\left( \% \right) = \frac{{{\text{I}}_{{\text{t}}} - {\text{I}}_{{\text{c}}} }}{{{\text{I}}_{0} - {\text{I}}_{{\text{c}}} }} \times 100\% ,$ where I_t_, I_0_ and I_c_ are the relative fluorescence intensity of FAM from G^FAM^-NK cells at different incubation times, the initial relative fluorescence intensity of FAM of the G-NK cells and the relative fluorescence intensity of NK cells (control group), which were calculated by FACS.

To evaluate the specific targeting ability of G-NK cells, NK cells, R-NK cells and G-NK cells were labeled with ICG for cell tracking. HepG2 cells or HeLa cells were seeded into Petri dishes at 37 °C in a humidified incubator containing 5% CO_2_ for 24 h. NK^ICG^ cells, R-NK^ICG^ cells or G-NK^ICG^ cells were added to the HepG2 cells or HeLa cells at the E/T ratio of 10:1 for 1 h. Of coculture afterward, the medium was discarded, and the cells were washed with PBS twice to remove unattached NK cells. Then, the tumor cells were cultured for another 24 h and imaged by CLSM, and the cytotoxicity of G-NK cells was analyzed by FCM following our previously published protocols [26].

### Quantification of cytokine secretion in vitro and in vivo

To assess the cytokines secreted by G-NK cells in vitro, G-NK cells were cocultured with HepG2 cells as previously described, and the concentrations of TNF-α, IL-2 and IFN-γ were analyzed by ELISA kits according to the manufacturer’s protocols. To evaluate the intratumoral cytokine levels, 0.02 g of tumor tissue was homogenized, and the concentrations of TNF-α, IL-2, perforin and IFN-γ were analyzed by ELISA kits according to the manufacturer’s protocols.

### G-NK cells enhance the therapeutic efficacy of ACT for GPC3 + tumors

To evaluate the specific targeting ability of G-NK cells, nude mice (male, BALB/c, 4–5 weeks) with body weights of ~ 20 g were subcutaneously injected with HepG2 cells (5 × 10^6^ cells) in sterilized PBS to establish a subcutaneous tumor model according to previously published works with slight modifications [7]. After 14 days, when the volumes of tumors reached ~ 70 mm^3^, 5 × 10^6^ G-NK cells, R-NK cells or NK cells were intravenously injected into each mouse (n = 4). The same volume of PBS was injected as a control. The mice were divided into the following 4 groups and received the following treatments:

1. Intravenous injection of 100 μL PBS for a total of 3 times per 3 days (n = 4);
2. Intravenous injection of 100 μL PBS containing 5 × 10^6^ NK cells with 1000 U/mouse IL-2 for a total of 3 times per 3 days (n = 4);
3. Intravenous injection of 100 μL PBS containing 5 × 10^6^ R-NK cells with 1000 U/mouse IL-2 for a total of 3 times per 3 days (n = 4);
4. Intravenous injection of 100 μL PBS containing 5 × 10^6^ G-NK cells with 1000 U/mouse IL-2 for a total of 3 times per 3 days (n = 4).

The tumor volumes and body weights of the mice in each treatment group were measured by Vernier calipers and electronic balance every 2 d, respectively. Tumor volume was calculated using the following equation:

1 $${\text{V}} = {\text{X}}*{{\text{Y}}^2}/2$$

Where X and Y are the longer and shorter diameters (mm) of the tumor, which were measured with calipers. The maximal tumor size should not excessed 1000 mm^3^ according to our animal ethics committee, and mice were euthanized when the tumor volume exceeded 1000 mm^3^.

### Toxicity assessment and H&E staining

To assess potential side effects, the body weight of each mouse was recorded. To evaluate the long-term systematic toxicity to the major organs, the mice were sacrificed at 16 days after receiving immunotherapy, and the major organs and tumors were isolated and sliced for staining with H&E according to our previously reported protocols [7]. Images were recorded with a Zeiss microscope (Axio Lab.A1).

### Statistical analysis

Statistical analysis of data was performed using one-way of variance (ANOVA) or Student’s t tests, *p < 0.05, **p < 0.01, ***p < *0.001.* All data are shown as the means ± SD of at least three experiments.

## Results

### Generation and Characterization of G-NK cells

**Fig. 1:**
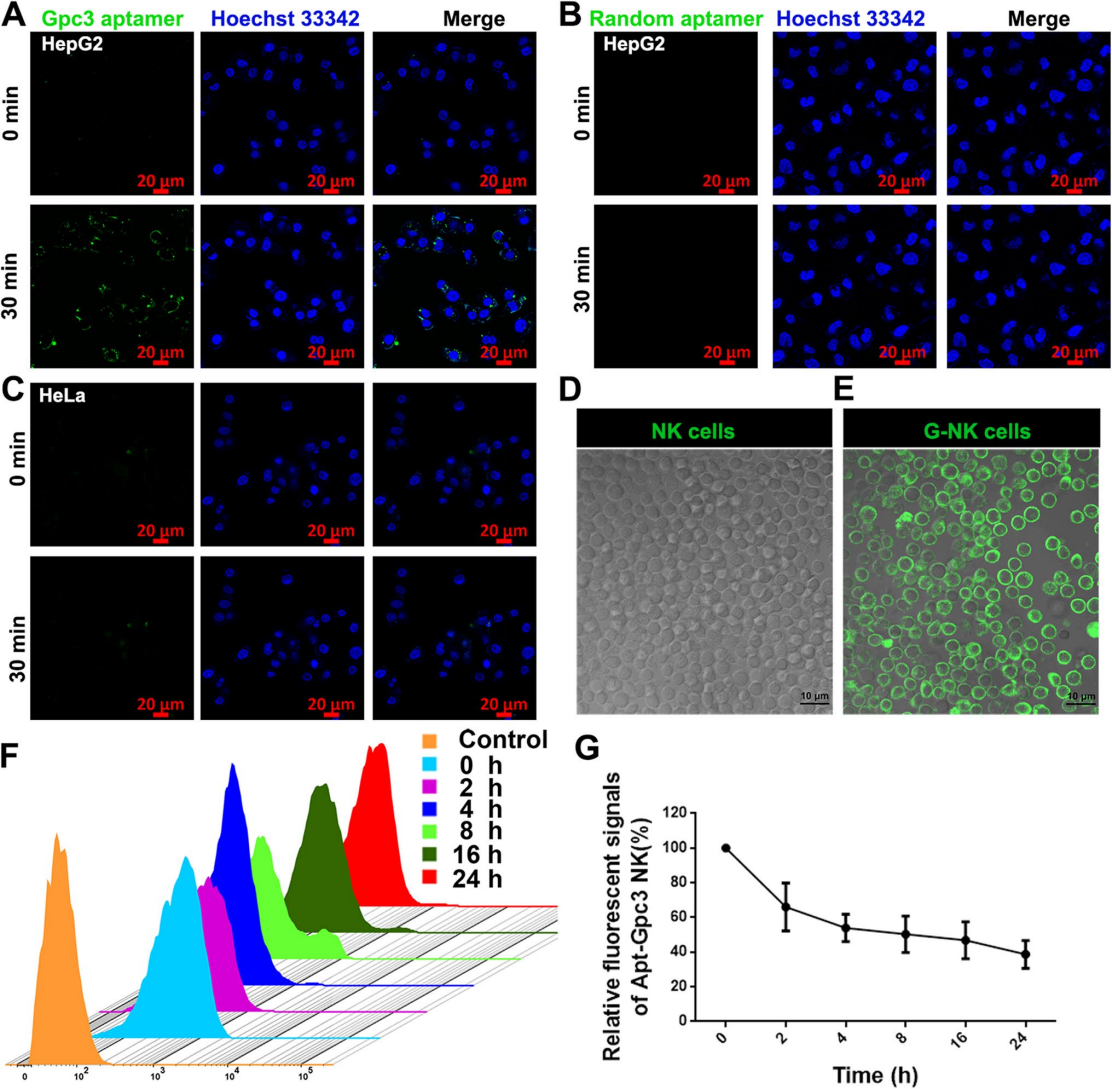
The characterization of Gpc3 aptamer and G-NK cells. **A**, **B** Confocal microscopy (CLSM) image of HepG2 cells after incubation with Gpc3 aptamer^FAM^ (**A**) and random aptamer (**B**) for 30 min. Scale bar, 20 μm. **C** CLSM image of HeLa cells after incubation with Gpc3 aptamer^FAM^ for 30 min. Scale bar, 20 μm. **D**, **E.** CLSM images of NK cells and G^FAM^-NK cells. Scale bar, 10 μm. **F** The dynamic stability of Gpc3 aptamer^FAM^ labelled on NK cells. **G** The relative fluorescence intensity of FAM from G^FAM^-NK cells at different incubation times, n = 3. G-NK cells without in vitro culture were defined as 0 h group in F-G

First, to endow NK cells with the ability to specifically target GPC3, the free Gpc3 aptamer must be able to target GPC3^+^ tumor cells but not control cells. To verify the targeting specificity of the Gpc3 aptamer, the binding ability between FAM dye-modified Gpc3 aptamers (Gpc3 aptamer^FAM^) (Supplemental Table S1) and the GPC3 protein on the surface of GPC3-overexpressing HepG2 cells was investigated, and the HeLa cells were used as the negative control. Western blot and FCM results first confirmed the overexpression of the GPC3 protein on HepG2 cells but not on HeLa cells (Supplemental Fig. S1A–C). Then, to investigate the specific targeting provided by the Gpc3 aptamers, HepG2 cells were imaged by confocal laser scanning microscopy (CLSM) after coincubation with Gpc3 aptamer^FAM^ for 30 min. Green fluorescence from Gpc3 aptamer^FAM^ was clearly observed on the surface of HepG2 cells (Fig. 1A). In contrast, the aptamer with a random Gpc3 mutation (Supplemental Table S1) failed to bind HepG2 cells (Fig. 1B). No obvious green fluorescence was detected in HeLa cells after 30 min of incubation, indicating that the Gpc3 aptamer^FAM^ failed to bind to HeLa cells (Fig. 1C). These results demonstrated the rapid and specific binding ability of the Gpc3 aptamer toward the GPC3 protein.

To endow NK cells with tumor-specific targeting properties, the Gpc3 aptamers were modified on the surface of NK cells by metabolic engineering following our previously reported protocol [28]. N-Azidoacetylmannosamine (Ac_4_ManNAz, 50 μM) was coincubated with NK cells for 48 h, and the cell viability assay confirmed that Ac_4_ManNAz labeling was nontoxic to NK cells (Supplemental Figs.s S2A, S3A and C). Afterward, dibenzylcyclooctyne-labeled Gpc3 aptamers (DBCO-Gpc3 aptamer) were covalently conjugated with the azido residues on the NK cell surface via a click reaction to generate G-NK cells. To facilitate the tracking of G-NK cells, the fluorescent dye FAM was used to label the DBCO-Gpc3 aptamer (DBCO-Gpc3 aptamer^FAM^), and its presence was verified by CLSM. As shown in Fig. 1E, strong green fluorescence signals were observed on the surface of G-NK cells compared to the untreated NK cells, indicating the successful conjugation of DBCO-Gpc3 aptamer^FAM^ on the surface of NK cells. The dynamic stability of G-NK cells was investigated by flow cytometry (FCM) to further validate the stability of Gpc3 aptamer modification. As shown in Fig. 1F and G, the FCM results showed that more than 42.04% of the G-NK cells retained relatively stable signals for up to 24 h. Furthermore, to evaluate whether modification with aptamers would have any influence on the characteristics of NK cells, activated NK cell biomarkers, including the CD3/CD56 or CD3/CD16, were investigated. The FCM analysis results showed that the coexpression patterns of CD3^−^/CD56^+^ (98.5%) and CD3^−^/CD16^+^ (89.5%) on G-NK cells were similar to those on unmodified NK cells (Supplemental Fig. S4). In addition, the viability of NK cells after modification with the Gpc3 aptamer was evaluated by CCK8 and Annexin V/PI assays. Compared to the parental NK cells without modification, G-NK cells showed no obvious change in cell status (Supplemental Fig. S2B). In addition, the FCM results confirmed that more than 90% of the G-NK cells were viable (Supplemental Figs. S3B and S3D). These findings revealed that the Gpc3 aptamer was successfully attached to the NK cell surface and remained stable for at least 24 h in vitro.

### The specific targeting property of G-NK cells in vitro

**Fig. 2:**
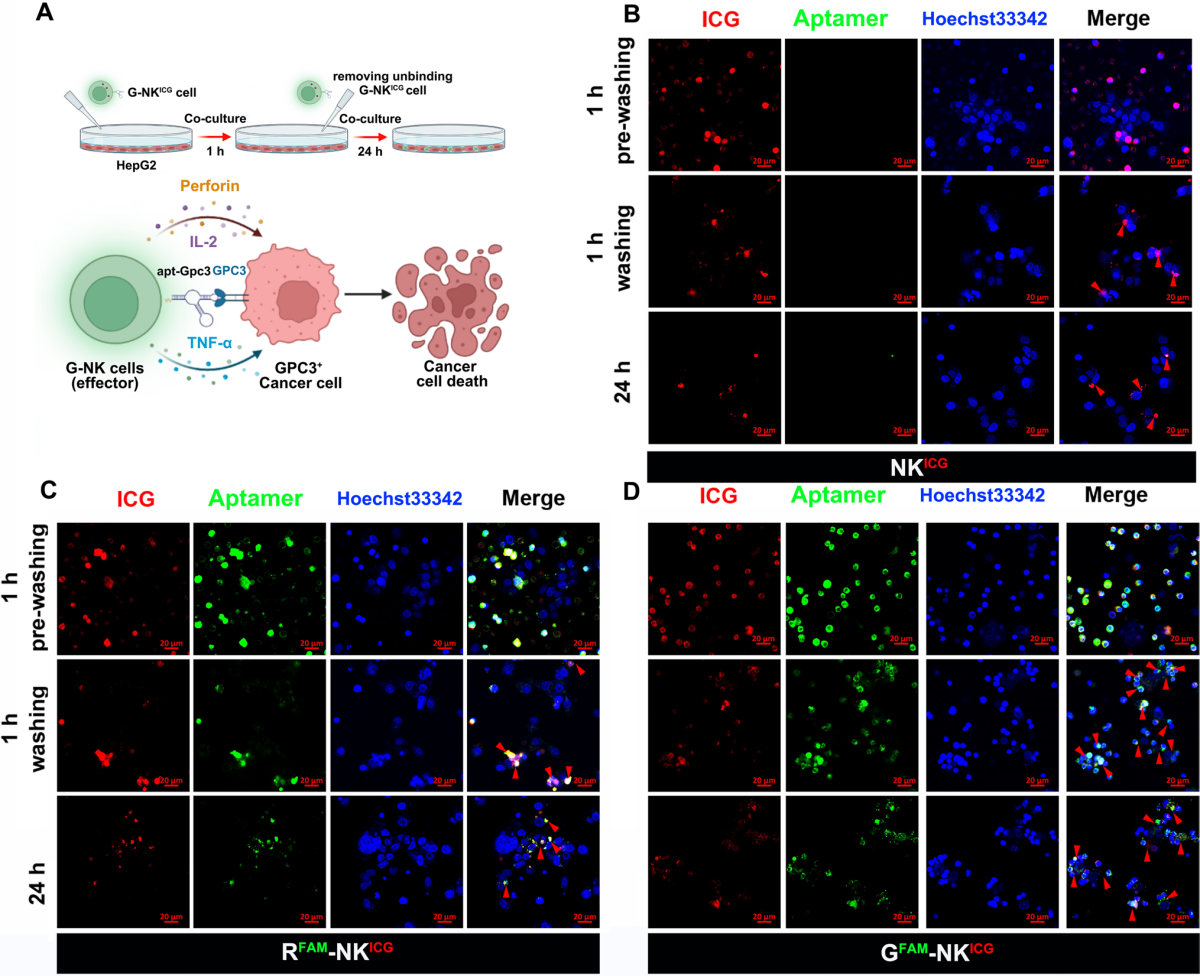
The characterization of G-NK cells. **A** Schematic illustration of the specific targeting property of G-NK cells. **B–D** CLSM image of HepG2 cells treated with NK^ICG^ cells (B), R^FAM^-NK^ICG^ cells (C) and G^FAM^-NK^ICG^ cells (D) for 1 h, washed twice with PBS, and then further incubated for 24 h at the E/T ratio of 10:1. Scale bar, 20 μm

To verify whether the G-NK cells could specifically target tumor cells, NK cells, random aptamer-modified NK cells (R-NK cells) and G-NK cells were coincubated with HepG2 and HeLa cells at an effector-to-target (E/T) ratio of 10:1. After 1 h of coincubation, the treated cancer cells were washed twice with PBS to remove the unattached NK cells, and the treated cells were cultured for another 24 h (Fig. 2A**)**. NK cells, R-NK cells and G-NK cells were labeled with ICG in this study for CLSM imaging. As shown in Fig. 2B–D, more red and green fluorescence was observed in the G-NK cell group at 1 h after washing and at 24 h, indicating that a large number of G^FAM^-NK^ICG^ cell clusters were attached to the HepG2 cell surface compared with NK^ICG^ cells and R^FAM^-NK^ICG^ cells. In contrast, no obvious differences of G-NK cell in HeLa cells were observed after washing compared with R-NK cell and NK cell-treated HeLa cells under the same conditions (Supplemental Figs. S5–S7), confirming the tumor cell-specific targeting ability of our modified cells. In addition, to further confirm the specific binding between G-NK cells and HepG2 cells, NK cells and HepG2 cells were stained with CM-DiL and Hoechst 33,342, respectively. Then, the HepG2^Hoechst 33342^ cells were digested to form a single-cell suspension and further coincubated with NK^CM−DiL^ cells, R-NK^CM−DiL^ cells or G-NK^CM−DiL^ cells at room temperature for 30 min. The cell mixtures were then directly analyzed by FACS. As shown in Supplemental Fig. S8, the percentages of fluorescence signals that overlapped (CM-DiL and Hoechst 33,342) were 65.2% in the G-NK cell group, 29.6% in the NK cell group and 31.6% in the R-NK cell group, implying that G-NK cells had better binding properties. These findings indicated that the prepared G-NK cells specifically targeted GPC3^+^ overexpressing HepG2 cells.

### G-NK cells show specific cytotoxicity toward GPC3 + tumor cells

**Fig. 3:**
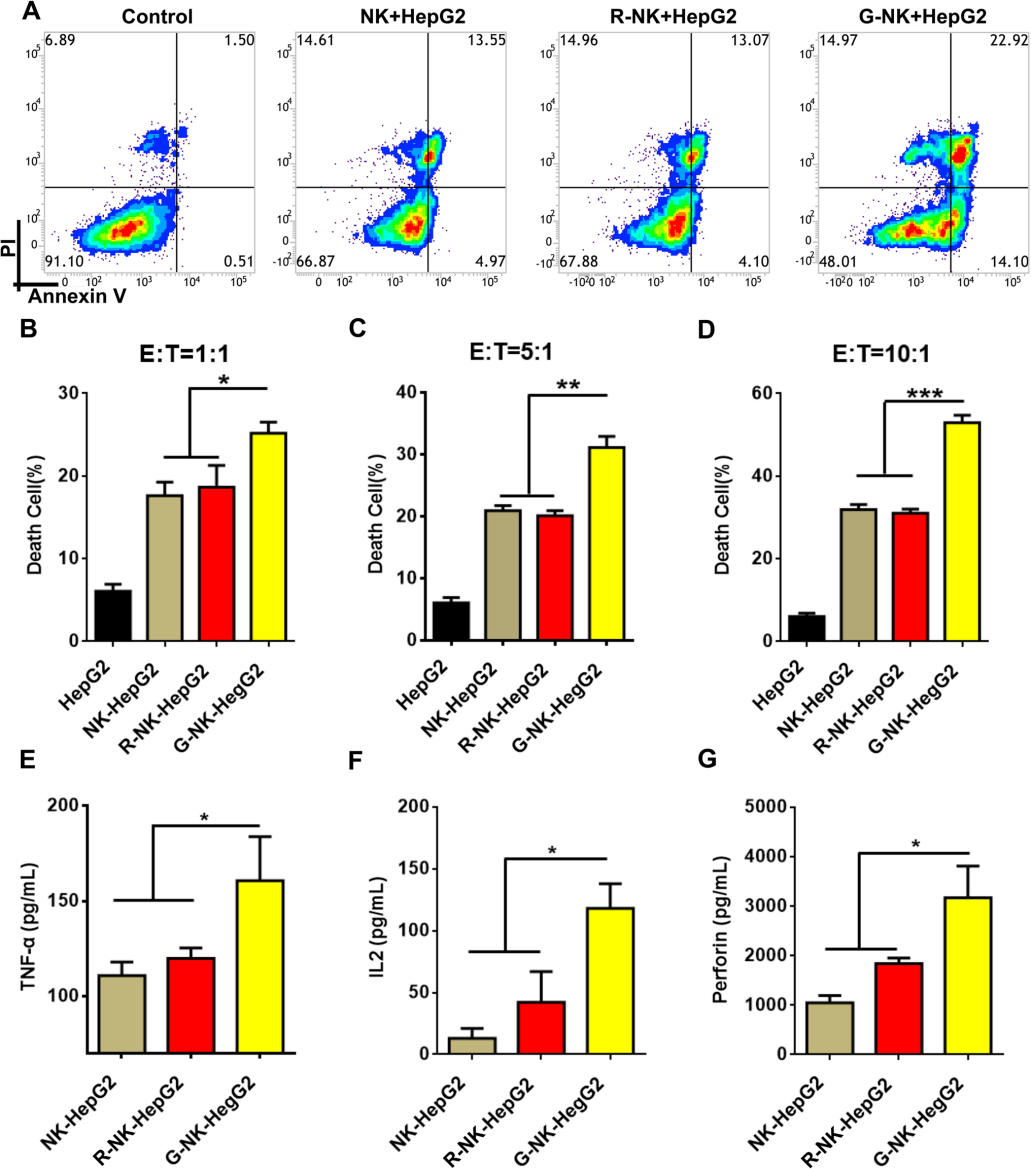
Cytotoxicity of G-NK cells toward GPC3^+^ Tumor cells. **A** Apoptosis/necrosis of HepG2 cells incubated with NK, R-NK and G-NK cells for 1 h and washed twice to remove unbinding NK cells, and then further incubated for 24 h at the E/T ratio of 10:1. **B**–**D** Apoptosis/necrosis of HepG2 cells incubated with NK, R-NK and G-NK cells at various of E/T ratio, n = 3. **E–H** The cytokine secretion from NK, R-NK and G-NK cells after co-incubating with HepG2 cells determined by ELISA analysis. Statistical analysis was performed by two-tailed paired sample Student’s t-tests (*p < 0.05, **p < 0.01, ***p < *0.001*), n = 3

To quantify cell apoptosis/necrosis mediated by G-NK cells, HepG2 cells and HeLa cells were coincubated with NK cells, R-NK cells or G-NK cells at the E/T ratio of 10:1, as indicated in Fig. 2A. The apoptosis/necrosis of the treated tumor cells was then analyzed by FCM after Annexin V-APC/propidium iodide (PI) staining. As shown in Fig. 3 A and D, more than 91.10% of the untreated HepG2 cells were viable. After coincubation with NK cells or R-NK cells, the apoptosis/necrosis rate of HepG2 cells increased to 31.85% in the NK cell-treated group and 32.12% in the R-NK cell-treated group. Interestingly, after coincubation with G-NK cells, the percentage of HepG2 cells that underwent apoptosis/necrosis significantly increased to 52.42%, indicating the enhanced cytotoxicity of G-NK cells mediated by the Gpc3 aptamer. As shown in Fig. 3B–D, after mixing HepG2 cells with G-NK cells at various E/T ratios and then removing the unbound G-NK cells with PBS, the rate of apoptosis/necrosis in HepG2 cells treated with G-NK cells markedly increased to 24.47%, 30.24% and 52.42% at E/T ratios of 1:1, 5:1 and 10:1, respectively. The cytotoxicity of G-NK cells was further confirmed with an LDH cytotoxicity assay. As shown in Figure S9, 38.76% of the HepG2 cells treated with G-NK cell treated HepG2 cells were viable, which was much lower than that of R-NK-treated HepG2 cells (54.12%) or NK cell-treated HepG2 cells (57.90%) under the same conditions, suggesting that the cytotoxicity of G-NK cells was significantly higher than that of the R-NK cells and NK cells. In addition, there were no significant changes in the rate of HeLa cells apoptosis/necrosis after coincubation with NK cells, R-NK cells and G-NK cells (Supplemental Figure S10A and S10B). The same results were observed in GPC3^+^ (Hep3B cell) and GPC3^−^ (Huh7 cell) lines (Supplemental Figure S11 and S12). These findings suggested that the remarkable cytotoxicity of G-NK cells was mediated by aptamer-specific recognition, which brings NK cells in close proximity to GPC3^+^ tumor cells to facilitate and prolong the interactions between the effectors and target cells.

After cell-to-cell contact, NK cells can also regulate adaptive immune responses through the secretion of immunoregulatory cytokines [2]. As shown in Fig. S13, the cytokines secreted by NK cells co-culturing with tumor cells were much higher than NK cells alone. Furthermore, to investigate whether Gpc3 aptamer could influence cytokine secretion from NK cells after binding with HCC cells, we evaluated the secretion of TNF-α, IL-2 and perforin after mixing HepG2 cells with NK cells, R-NK cells and G-NK cells at an E/T ratio of 10:1. As shown in Fig. 3C, significant upregulation of TNF-α, IL-2 and perforin was observed after treatment with activated G-NK cells compared to treatment with NK cells and R-NK cells. These results suggested that G-NK cells could specifically bind to GPC3^+^ tumor cells and upregulate the secretion of cytokines to activate the killing capabilities of NK cells in vitro.

### G-NK cells enhance GPC3 + tumor killing in vivo

**Fig. 4:**
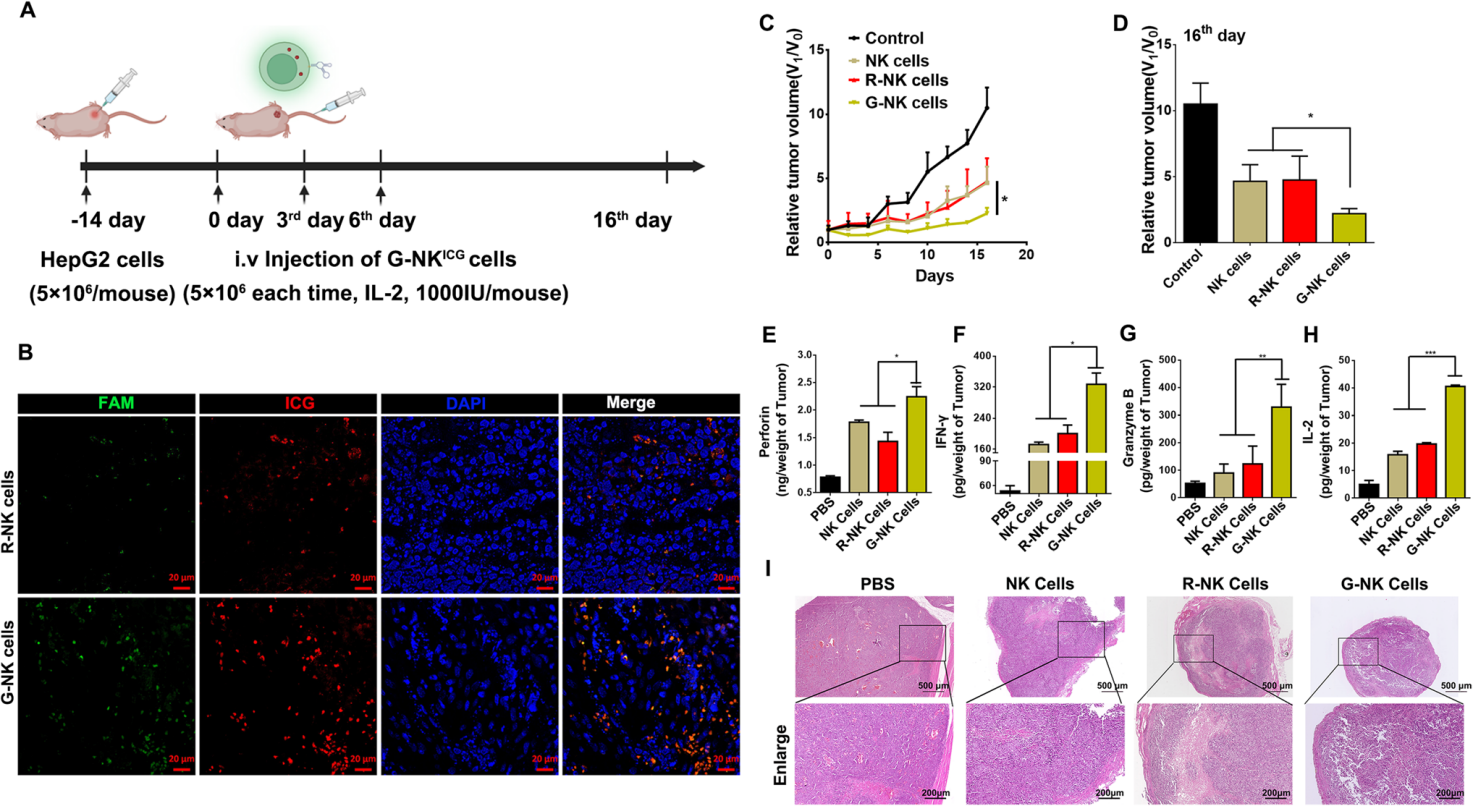
**A** Schematic illustration of the treatment protocol. **B** CLSM image of tumor slices after administration of R-NK cells or G-NK cells as indicated after 24 h. **C** The relative tumor volumes for mice treated as indicated. V_1_ is the final tumor volume and V_0_ is the initial tumor volume. Statistical analysis was performed with ANOVA (n = 4; *p < 0.05, **p < 0.01, *** p < *0.001*). **D** Ex vivo tumors in each group at 16 day after receiving various treatments as indicated. Statistical analysis was performed with ANOVA (n = 4; *p < 0.05, **p < 0.01, *** p < *0.001*). **E–H** Cytokine secretion from NK cells in tumors. Statistical analysis was performed with ANOVA (n = 3; *p < 0.05, **p < 0.01, *** p < *0.001*). **I** H&E staining of tumors after receiving different treatments. Scale bar: 500 μm, 200 μm

Encouraged by the specific targeting ability and high level of cytokine secretion from G-NK cells, we next investigated the specific targeting property of G-NK cells in vivo. As shown in Fig. 4A, a HepG2 tumor-bearing nude mouse model was established by subcutaneously injecting 5 × 10^6^ HepG2 cells into each mouse. When the tumor volume reached approximately 70 mm^3^, NK^ICG^ cells, R^FAM^-NK^ICG^ cells or G^FAM^-NK^ICG^ cells were intravenously injected into the mice. To investigate the biodistribution and accumulation of G-NK cells in the tumors, the major organs and tumors were dissected and imaged 24 h post-injection by ChemiDoc™ MP Image system and the mean fluorescence intensity were calculated by Image J. As shown in Supplemental Figure S14A and S14B, compared with the mice treated with NK^ICG^ cells or R^FAM^-NK^ICG^ cells, the G^FAM^-NK^ICG^ cell-treated mice showed relatively higher fluorescence signals in tumors, suggesting the active targeting ability of our prepared G-NK cells. To further confirm the accumulation of G^FAM^-NK^ICG^ cells in tumors, R^FAM^-NK^ICG^ cell- or G^FAM^-NK^ICG^ cell-treated mice were also sacrificed at 24 h, and the tumors were sliced and imaged by CLSM for ex vivo examination. As shown in Fig. 4B, compared to R^FAM^-NK^ICG^ cell-treated mice, the tumor tissue from G^FAM^-NK^ICG^-treated mice showed significantly higher red (ICG) and green (FAM) fluorescence signals with deeper penetration into the tumors. These results demonstrated that G-NK cells had excellent active targeting ability and could deeply penetrate into GPC3^+^ tumors, suggesting their promising potential to enhance ACT in GPC3^+^ solid tumors.

We next evaluated the adoptive immunotherapeutic effect of G-NK cells in vivo. When the tumor volume reached approximately 70 mm^3^, NK cells, R-NK cells or G-NK cells (5 × 10^6^ cells per mouse) were administered to tumor-bearing mice and supplemented with IL-2 (1000 units) on Days 0, 3 and 6 (Fig. 4A). All three types of NK cells inhibited tumor growth compared with the PBS control (Fig. 4C, D). G-NK cells showed the most remarkable, significant inhibitory effect when compared with the other two groups (administered with NK cells and R-NK cells) on the 16th day. To further evaluate the cytotoxic effect of NK cells in vivo, cytokine secretion from NK cells in tumors was analyzed by ELISA. As shown in Fig. 4E–H, compared with the groups treated with NK^ICG^ cells and R-NK^ICG^ cells, the G-NK^ICG^ cell-treated groups showed the highest amounts of cytokine secretion (such as perforin, granzyme B, IFN-γ and IL-2). To confirm the immunotherapeutic efficacy, dissected tumor tissues were analyzed by hematoxylin and eosin (H&E) staining. The results showed that the G-NK cell-treated group had a more obvious necrotic area with nuclear shrinkage than any other group (Fig. 4I). Taken together, the above results demonstrated that G-NK cells could enhance immunotherapeutic efficacy due to the specific GPC3^+^ tumor-targeting ability mediated by the Gpc3 aptamer.

### The potential toxicity of G-NK cells in vivo

**Fig. 5:**
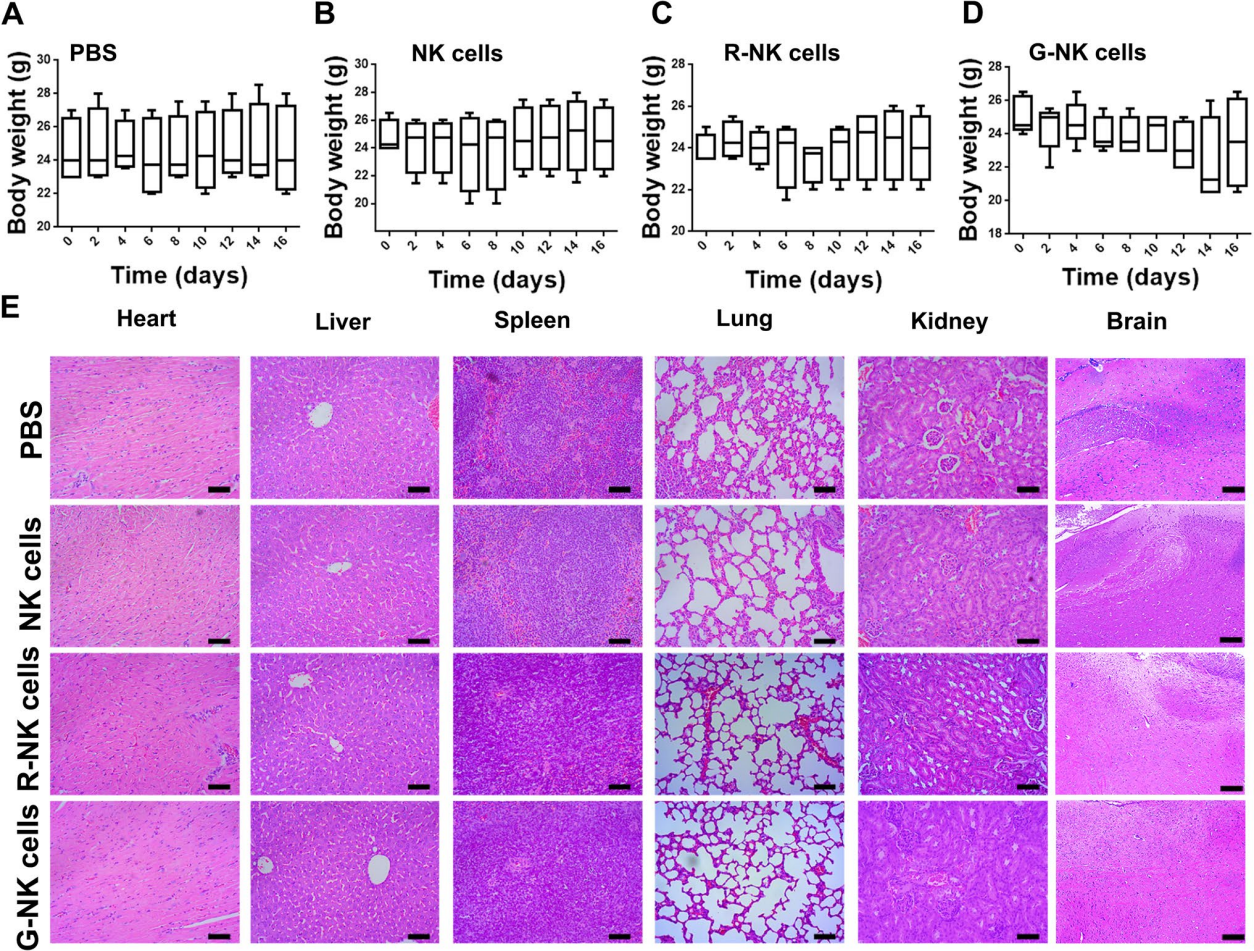
**A**–**D** The body weight change of mice after receiving different treatments as indicated. **E** H&E staining of major organs after receiving different treatments. Scale bar: 50 μm

Finally, the toxicity of G-NK cells was evaluated in vivo. The body weights of the mice that underwent adoptive transfer of NK cells, R-NK cells or G-NK cells were recorded. In addition, the major organs of the mice, including the spleen, liver, heart, lung, kidney and brain, were examined by H&E staining after receiving different treatments. As shown in Fig. 5A–D, no noticeable body weight loss was observed. Moreover, the H&E image showed that no obvious histological difference between different group (Fig. 5E). These findings suggested the safety of our G-NK cells in vivo.

## Discussion

The options for HCC treatment have considerably improved in the last few years and include surgical resection, TACE, liver transplantation and immune check-point inhibitors [29]. However, the clinical effects of these therapies are limited and the overall HCC mortality rate remains disturbingly high [30]. Therefore, developing novel strategies for the treatment of patients with HCC is urgently needed. CAR T or CAR NK cells possess special targeting properties and activation mechanisms that have been attributed to the specific structure of CAR [16, 31] and represent a complementary therapeutic option for HCC. Although CAR NK cells have shown promising results in preclinical studies [4], they still face obstacles, including the complex operation procedures, high costs and potential safety risks [14–16]. In the present work, NK cells were modified with the Gpc3 aptamer without viral infection. Moreover, G-NK cells are unable to be overactivated due to the lack of intracellular activation signaling pathways in the CAR structure. These data indicate that G-NK cells can be considered safer than CAR T or CAR NK cells.

The rational selection of scFv fragments for CAR is critical for effective clinical benefit. Several tumor associated antigens (TAAs) expressed in liver tumor cells have been exploited for CAR-T cell therapy, including GPC-3 [32], AFP [33], and CEA [34]. GPC-3 is highly expressed in 56.3% of HCC serum and undetectable in healthy liver cells and other organs [27]. Moreover, the expression of GPC3 is maintained on HCC cell surface even after disease relapse [35]. Previously, GPC3-targeted CAR NK cells were reported to show toxicity to HCC cells [30]. Moreover, therapeutic approaches utilizing GPC3-targeted immunotherapy have also been shown to be safe [32]. These data indicate that GPC3, as a tumor associated antigens of HCC, is a reasonable target for ACT treatment.

DNA and RNA aptamers have attracted great attention as a new generation of therapeutics due to their various advantages [36]. The three-dimensional conformation of an aptamer allows it to bind various targets, such as small molecules, proteins and cells. Pegaptanib (an aptamer that antagonizes the action of VEGF) is the first FDA-approved aptamer [37], and our previous work reported that NK cells equipped with TLS11a and PD-L1 aptamer for targeting HepG2 cells and blocking the immunoinhibitory pathways of NK cells showed promising therapeutic efficacy [26]. In our work, the Gpc3 DNA aptamer possessed high specificity to bind to its specific target (the GPC3 protein). Based on our findings, NK cells modified with the Gpc3 aptamer could enhance the clinical therapeutic effect in HCC patients who were pathologically diagnosed with GPC3^+^ HCC by immunohistochemical staining. Moreover, the chemical synthesis of aptamers can standardized, providing an opportunity to meet a wide range of requirements during clinical applications.

In addition, anchoring of lipid-DNA aptamers to NK cells has been reported as an alternative nongenetic strategy [20]. Although this strategy endowed NK cells with specific targeting characteristics, the anchoring reaction was unstable and limited its applications. In the present work, DBCO-modified DNA aptamers could be anchored to the membranes of NK cells through metabolic glycoengineering via a click-on reaction, which is simple and highly efficient without genetic modification.

In summary, we developed a strategy to modify NK cells with Gpc3 aptamers (G-NK cells), which can specifically target GPC3^+^ HCC tumor cells to significantly enhance NK cell cytotoxicity without genetic modification in vitro and in vivo. The proposed strategy shows the following advantages. (i) The GPC3 aptamer could be synthesized easily to specifically target GPC3^+^ tumor cells; (ii) G-NK cells could be obtained simply and stably with low cost; (iii) G-NK cells specifically targeted GPC3^+^ HCC tumor cells and thereafter secreted more cytokines to preserve the intense cytotoxic activities of NK cells. It was found that GPC3 was highly expressed in the tumor tissues compared with the non-HCC tissues according to The Cancer Genome Atlas (TCGA) cohort (n = 369), the RNA-seq (n = 65) and MS data (n = 152) of Mengchao Hepatobiliary Hospital of Fujian Medical University (Supplemental Figure S15). Therefore, G-NK cells could be used as a potential immunotherapy for HCC patients who were diagnosed GPC3^+^ by immunohistochemistry; and (iv) The G-NK cells were biocompatible with no obvious in vivo cytotoxicity. Based on the above advantages, we believe that G-NK cells hold considerable potential for enhancing immunotherapy against GPC3^+^ tumors for future clinical translation.

## Ethics approval

All animal procedures were approved by the Animal Ethics Committee of Mengchao Hepatobiliary Hospital of Fujian Medical University. The maximal tumor size should not excessed 1000 mm^3^ according to our animal ethics committee. And mice were euthanized when the tumor volume exceeded 1000 mm^3^. We confirmed that the maximal tumor size was not exceeded 1000 mm^3^.

### Supplementary Information

Additional file 1 (DOCX 3396 KB)

## Data Availability

The data presented in this study are contained within the article.

## Abbreviations

ACT: Adoptive cell therapy
CAR NK cells: Chimeric antigen receptor NK cells
CD16: Cluster of differentiation 16
CLSM: Confocal laser scanning microscopy
FCM: Flow cytometry
GPC3: Glypican-3
HCC: Hepatocellular carcinoma
ICG: Indo Cyanine Green
IL-2: Interleukin-2
PD-L1: Programmed cell death ligand 1
R-NK cells: NK cells modified with random mutation Gpc3 aptamer
TNF-α: Tumor necrosis factor α
Ac_4_ManNAz: N-azidoacetylmannosamine
CD3: Cluster of differentiation 3
CD56: Cluster of differentiation 56
DBCO: Dibenzylcyclooctyne
G-NK cells: NK cells modified with Gpc3 aptamer
GZMB: Granzyme B
H&E staining: Hematoxylin and Eosin staining
IFN-γ: Interferon-γ
NK cells: Natural killer cells
PI: Propidium iodide
SELEX: Systematic evolution of ligands by exponential enrichment
