# Supplementary material for: Natural killer cells modified with a Gpc3 aptamer enhance adoptive immunotherapy for hepatocellular carcinoma
Source: Discov Oncol. 2023 Sep 4;14:164. doi: 10.1007/s12672-023-00780-6 (PMC10477160; doi:10.1007/s12672-023-00780-6)
Supplement: Supplementary file 1 — Additional file 1 (DOCX 3396 KB) [file 12672_2023_780_MOESM1_ESM.docx]

Supporting Information

Natural Killer Cells Modified with a Gpc3 Aptamer Enhance Adoptive Immunotherapy for Hepatocellular Carcinoma

Youshi Zheng^2,3^, Zisen Lai^2^, Bing Wang^2,3^, Zuwu Wei^2,3^, Yongyi Zeng^1,2,3^, Qiuyu Zhuang^2,3*^,Xiaolong Liu^2,3,4*^, Kecan Lin^1,2,4^*.

1. Liver Disease Center, The First Affiliated Hospital of Fujian Medical University, Fuzhou 350005, China.
2. The United Innovation of Mengchao Hepatobiliary Technology Key Laboratory of Fujian Province, Mengchao Hepatobiliary Hospital of Fujian Medical University, Fuzhou, P. R. China.
3. Mengchao Med-X Center, Fuzhou University, Fuzhou, P. R. China.
4. Fujian Provincial Clinical Research Center for Hepatobiliary and Pancreatic Tumors

*Corresponding authors: Qiuyu Zhuang, Xiaolong Liu & Kecan Lin

**Email**: qiuyuzhuang@outlook.com, xiaoloong.liu@gmail.com & kclin2002@126.com.

**Results**

**Table S1**. The detailed information of aptamer sequences

| Name | Sequence from 5’ to 3’ |
| --- | --- |
| DBCO-GPC3 aptamer | DBCO-TAACGCTGACCTTAGCTGCATGGCTTTACATGTTCCA-FAM |
| Random-DBCO-GPC3 aptamer | DBCO-TgAtGaTtAaCgTcGtTaCcTtGaTcTgCgTaTcCtA-FAM |

The minuscule letters represent mutations.

**Figure S1**

**
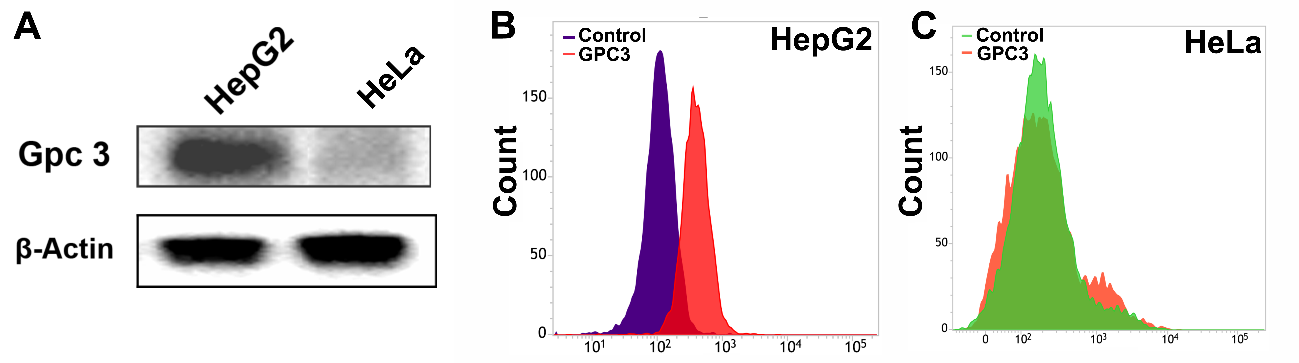
**

**Figure S1.** **A.** Western Blot for the expression of GPC3 protein in HepG2 cells and HeLa cells. **B-C.** The surface expression level of GPC3 on HepG2 (B) and HeLa cells (C) evaluated by FACS.

**Figure S2**


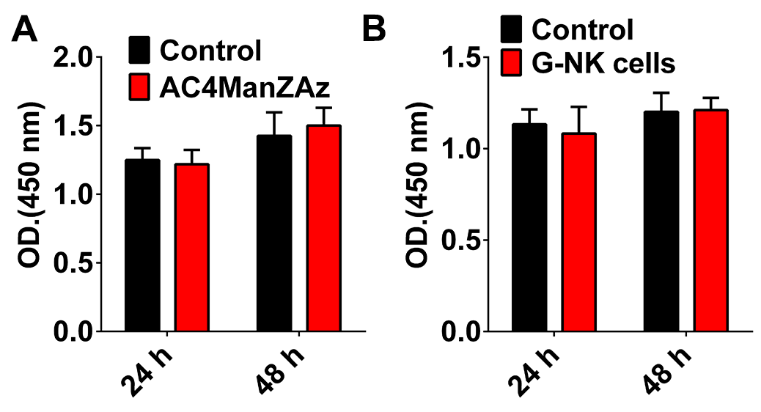


**Figure S2.** **A.** The viability of NK cells incubated with Ac4ManNAz for 24 h and 48 h, respectively. NK cells without Ac4ManNAz were served as the control group. **B.** The viability of G-NK cells for 24 h and 48 h. Analyzed by cell counting kit-8 (CCK8) assays. NK cells treated with Ac4ManNAz were served as the control group. The statistical analysis was performed with the ANOVA analysis (**p < 0.05, **p < 0.01*), the data are presented as the mean ± SD, n=3.

**Figure S3**

*
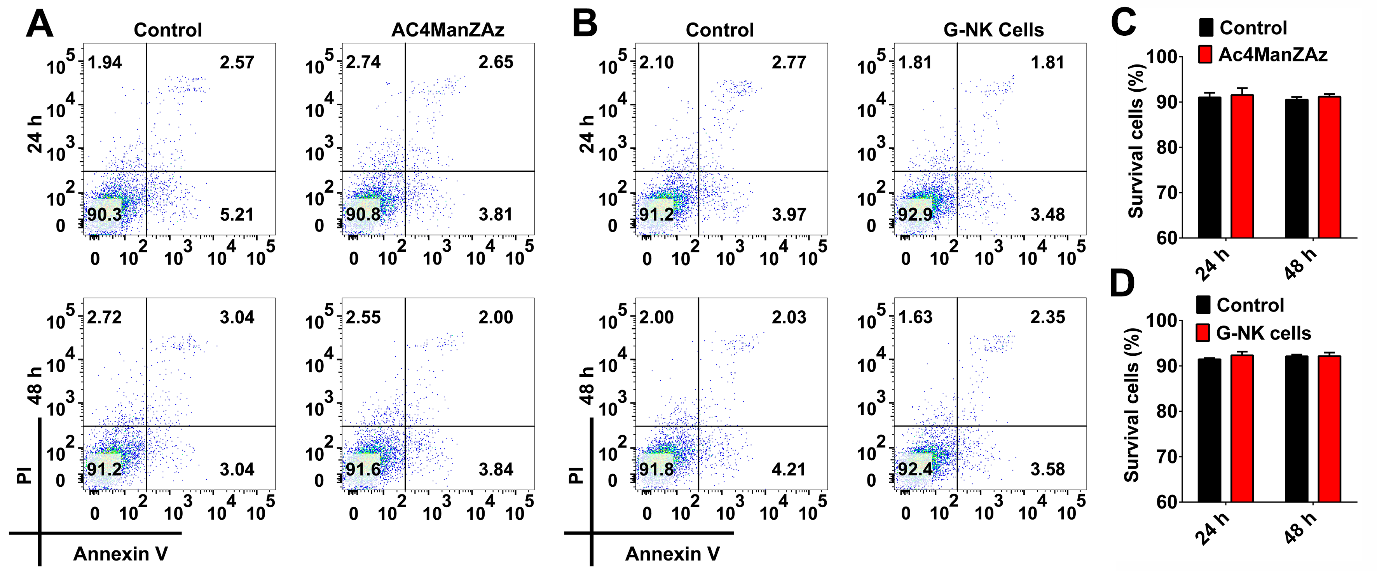
*

**Figure S3. A and C** The apoptosis/necrosis of NK cells incubated with Ac4ManNAz (50 μM) for 24 h and 48 h, respectively. NK cells without Ac4ManNAz were served as the control group. **B and D** The apoptosis/necrosis of G-NK cells for 24 h and 48 h. Analyzed by FACS. NK cells treated with Ac4ManNAz were served as the control group. The statistical analysis was performed with the ANOVA analysis (n=3,**p < 0.05, **p < 0.01*).

**Figure S4**


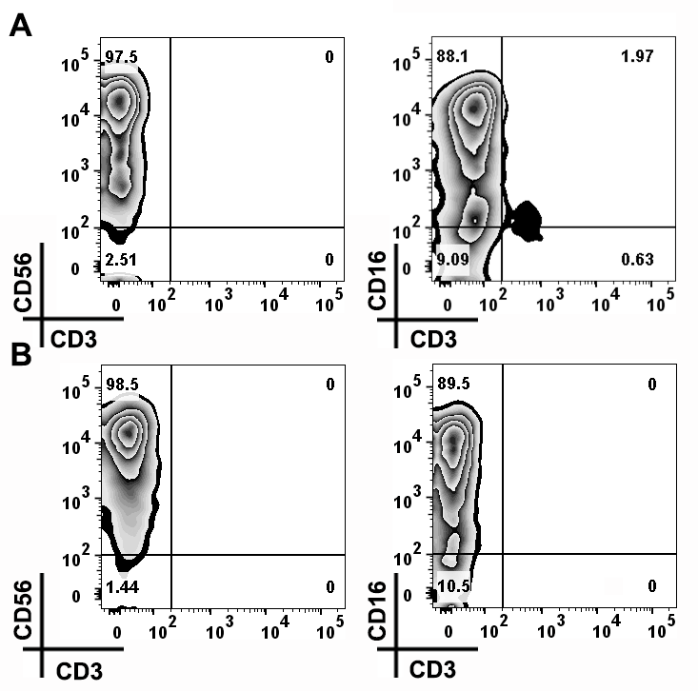


**Figure S4. A**. The activation of NK cells was analyzed by staining CD3^-^/CD56^+^ and CD3^-^/CD16^+^. **B.** The phenotype of G-NK cells treated for another 48 h.

**Figure S5**


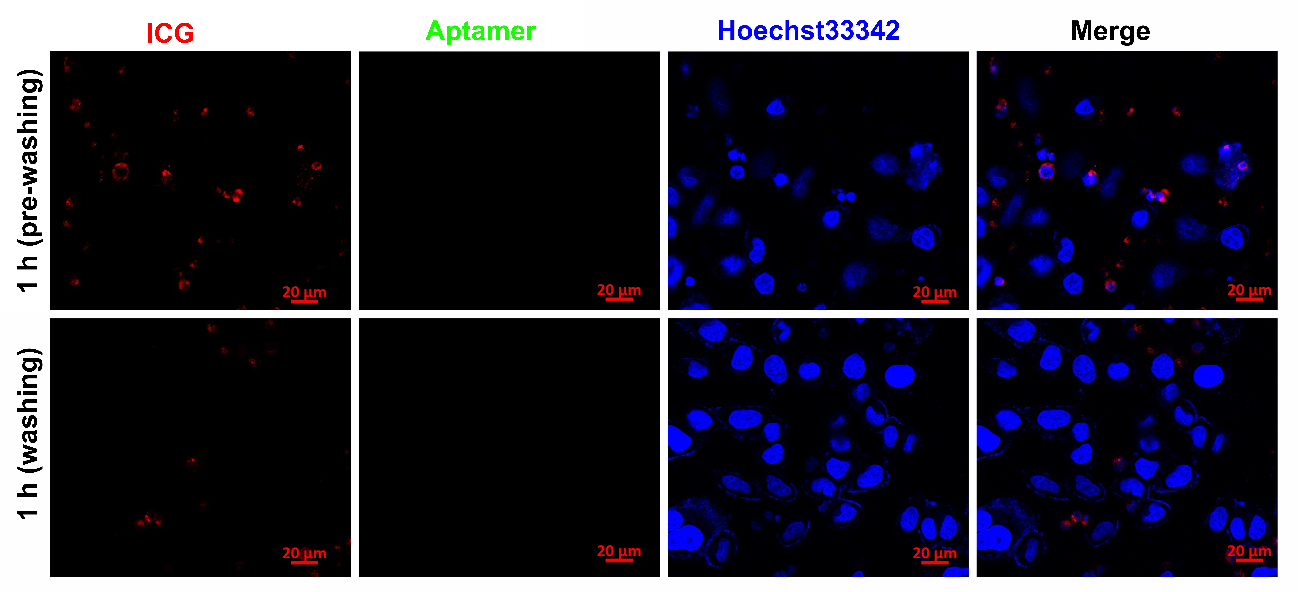


**Figure S5.** CLSM images of HeLa cells treated with NK^ICG^ cells for 1 h at the E/T ratio of 10:1 and then washing twice with PBS. Scale bar, 20 μm.

**Figure S6**


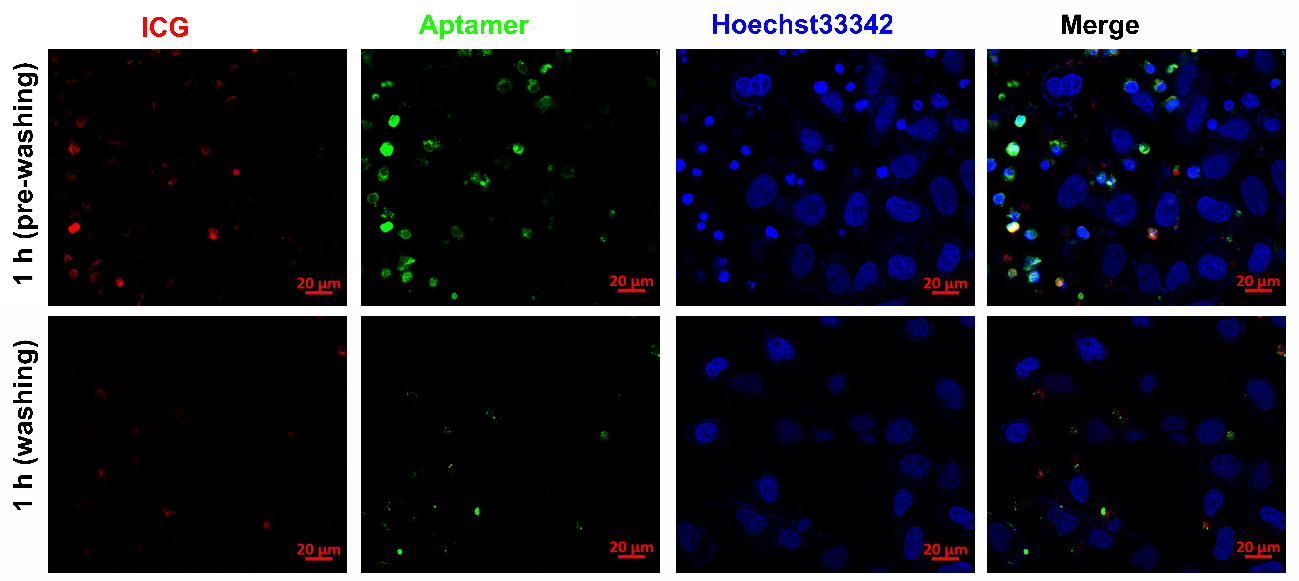


**Figure S6.** CLSM images of HeLa cells treated with R^FAM^-NK^ICG^ cells for 1 h at the E/T ratio of 10:1 and then washing twice with PBS. Scale bar, 20 μm.

**Figure S7**


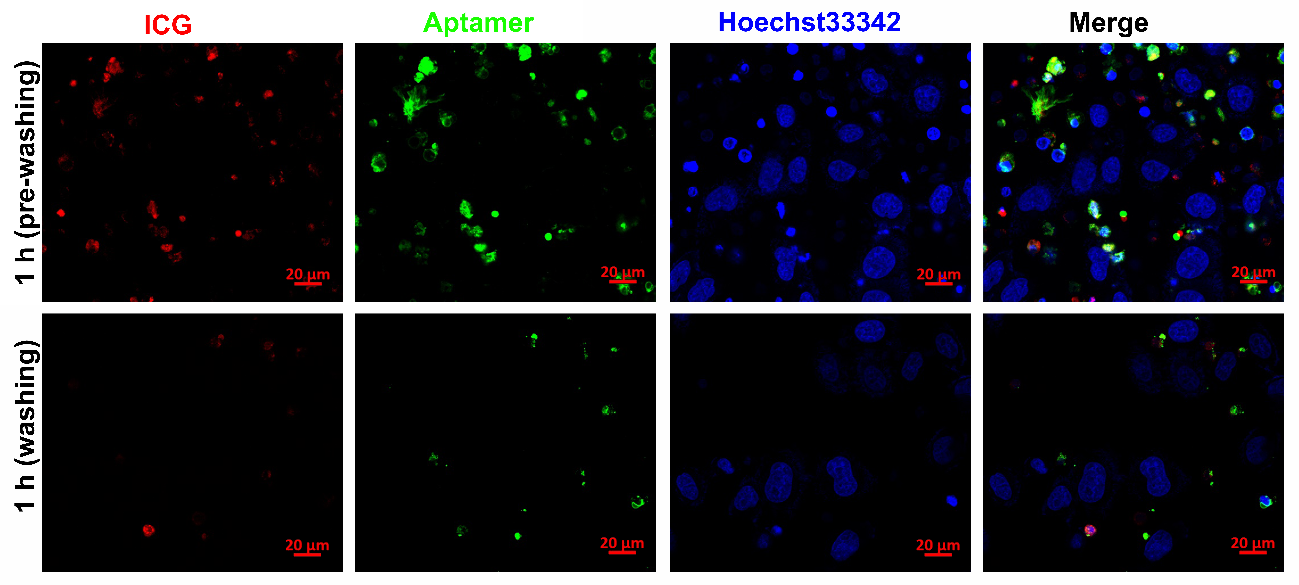


**Figure S7.** CLSM images of HeLa cells treated with G^FAM^-NK^ICG^ cells for 1 h at the E/T ratio of 10:1 and then washing twice with PBS. Scale bar, 20 μm.

**Figure S8**


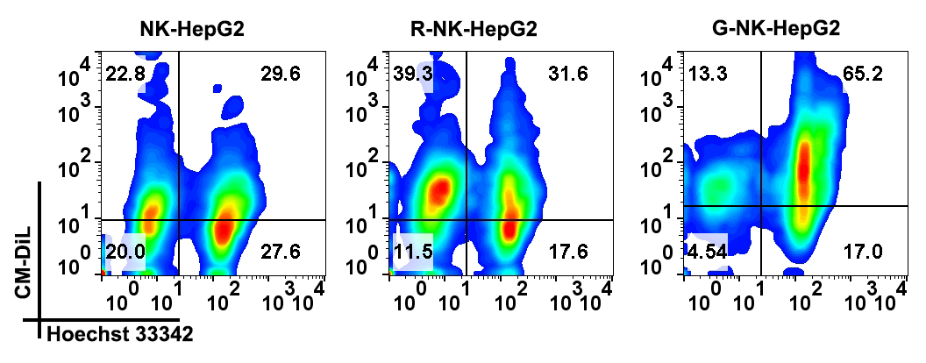


**Figure S8.** The specific targeting property of NK cells, R-NK cells and G-NK cells evaluated by FACS.

**Figure S9**


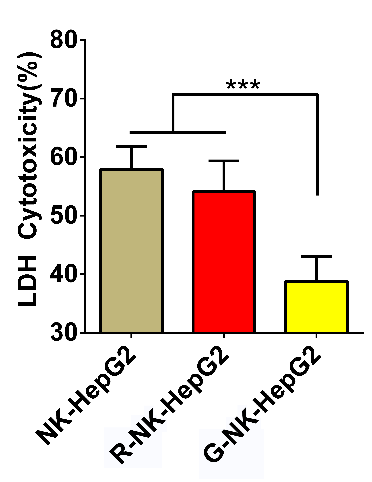


**Figure S9.**The cell viability of HepG2 cells when co-incubated with G-NK cells, R-NK cells or NK cells (E/T=10:1) evaluated by LDH cytotoxicity assay. The statistical analysis was performed with the ANOVA analysis (**p < 0.05, **p < 0.01*), the data are presented as the mean ± SD, n=5.

**Figure S10**


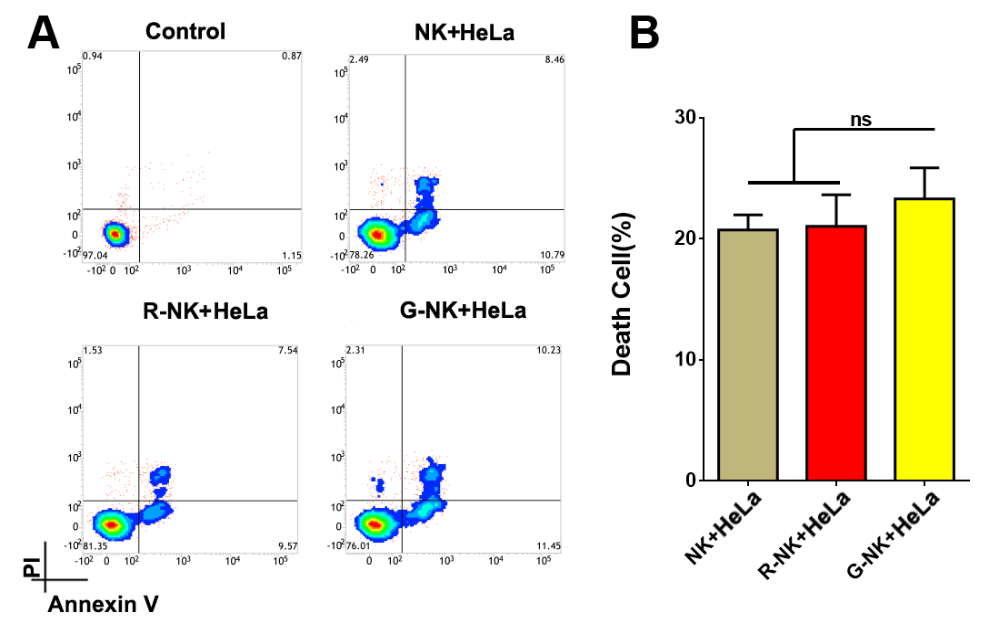


**Figure S10.** **Cytotoxicity of G-NK cells for GPC3^-^ Tumor cells.** **A.** Apoptosis of HeLa cells incubated with NK, R-NK and G-NK cells for 1h and washed twice to remove unbinding NK cells, and then further incubated for 24 h at the E/T ratio of 10:1. **B.** Apoptosis of HepG2 cells incubated with NK, R-NK and G-NK cells at E/T ratio of 10:1. The statistical analysis was performed with the ANOVA analysis (**p < 0.05, **p < 0.01*), the data are presented as the mean ± SD, n=3.

**Figure S11**

**
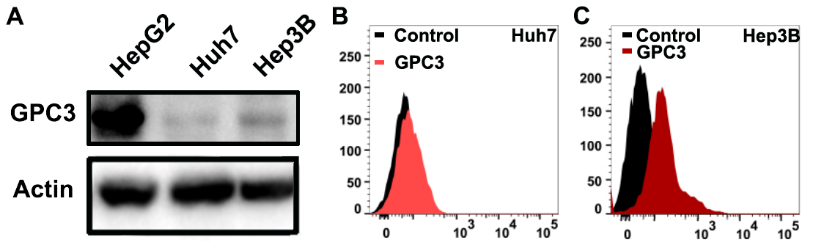
**

Figure S11. **A.** Western Blot for the expression of GPC3 protein in HepG2, Huh7 and Hep3B cells. **B-C.** The surface expression level of GPC3 on Huh7 (B) and Hep3B cells (C) evaluated by FACS.

**Figure S12**


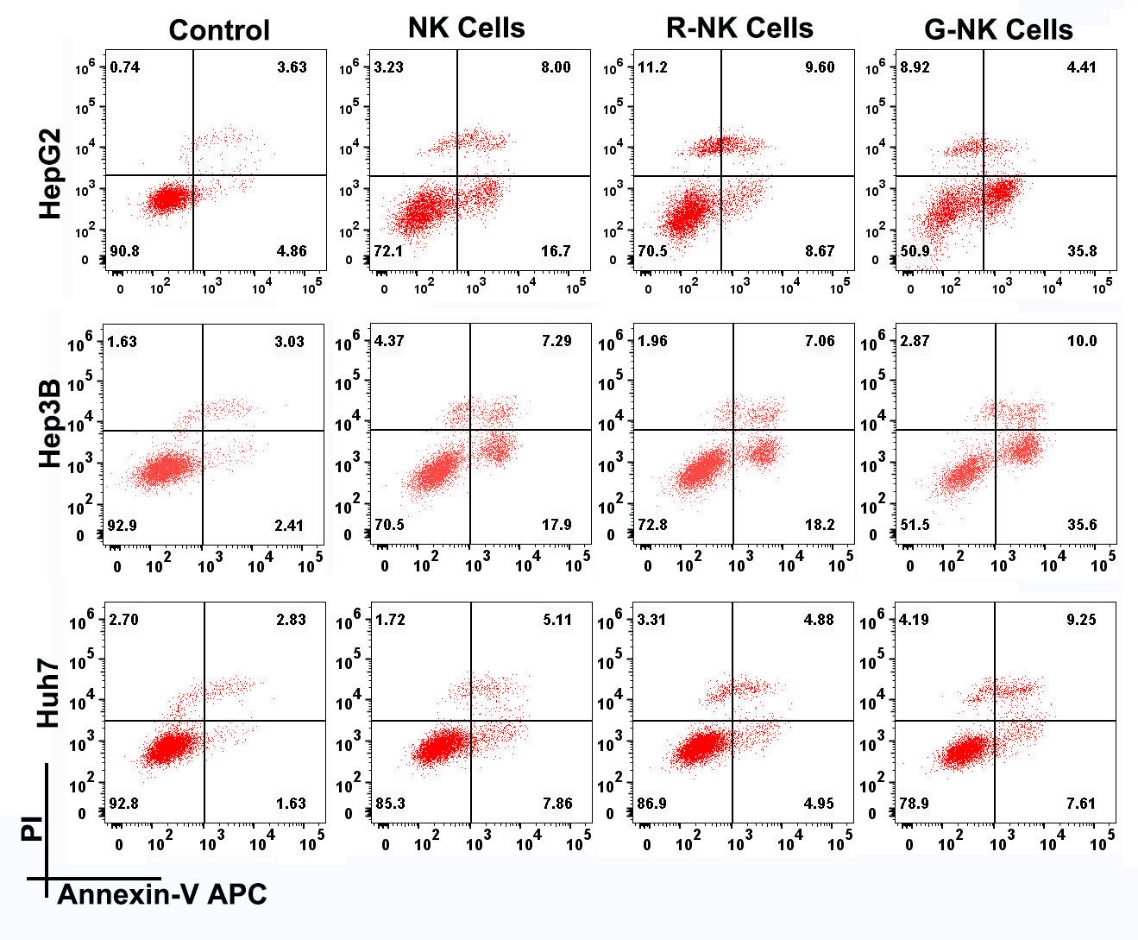


**Figure S12.** The cytotoxicity of G-NK cells toward HepG2, Hep3B and Huh7 cells. The HepG2, Hep3B and Huh7 cells when co-incubated with NK cells, R-NK cells and G-NK cells (E/T=10:1) for 1h and washed twice to remove unbinding NK cells, and then further incubated for 24 h evaluated by FACS.

**Figure S13**


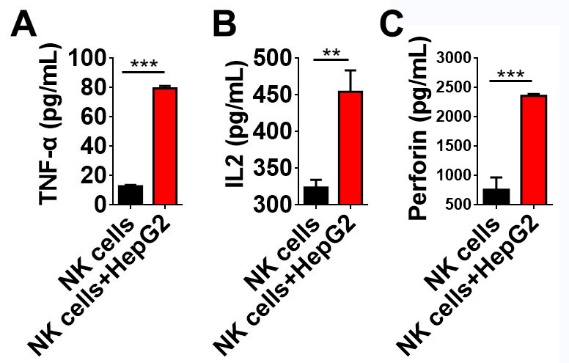


**Figure S13.** The cytokines secreted by NK cells with or without coincubating with HepG2 cells.

**Figure S14**


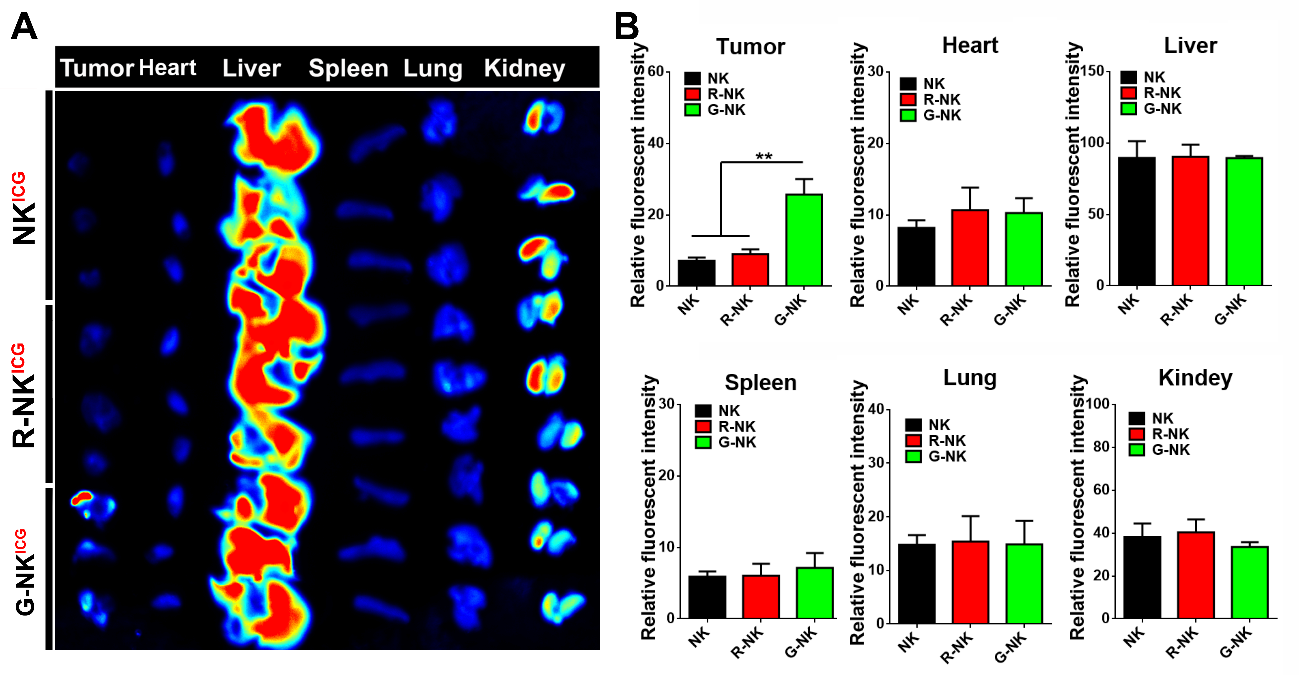


**Figure S14. A.** *Ex vivo* NIR fluorescence images of tumors and other major organs after treating with R-NK cells or G-NK cells for 24 h. ICG was excited by 808 nm. **B.** Relative fluorescence intensity of ICG in NK cells, R-NK cells or G-NK cells as indicated A. The fluorescence intensity was calculated by Image J. Statistical analysis was performed by two-tailed paired sample Student’s t-tests (**p < 0.05, **p < 0.01*), n = 3.

**Figure S15**


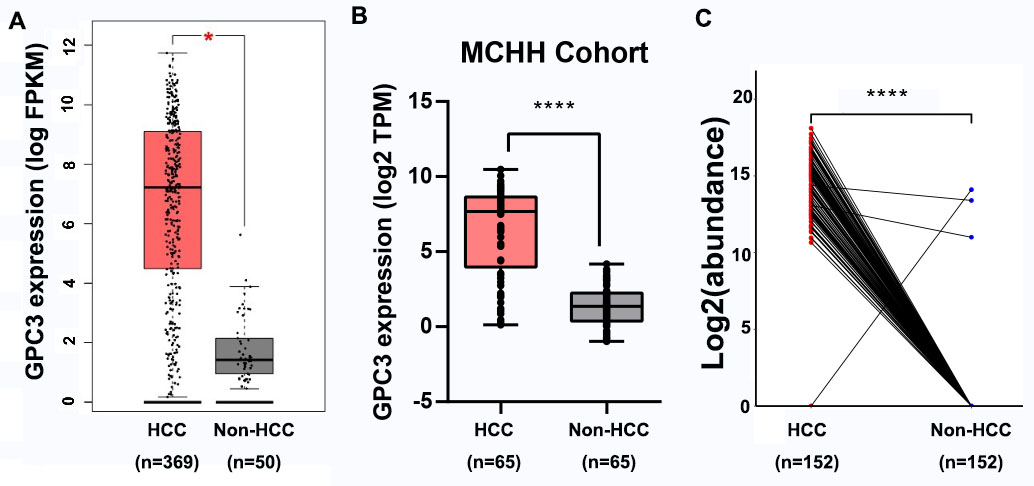


**Figure S15.** **A.**The expression of GPC3 in HCC tumours of TCGA cohort using the online TCGA analysis tool (http://gepia.cancer-pku.cn/). **p* < 0.05. **B-C.** The expression of GPC3 in HCC patients using RNA-seq and MS. *****p* < 0.0001.
